# Antioxidant Properties of *Lippia alba* Essential Oil: A Potential Treatment for Oxidative Stress-Related Conditions in Plants and Cancer Cells

**DOI:** 10.3390/ijms25158276

**Published:** 2024-07-29

**Authors:** Ilaria Borromeo, Anastasia De Luca, Fabio Domenici, Cristiano Giordani, Luisa Rossi, Cinzia Forni

**Affiliations:** 1 Department of Biology, University of Rome Tor Vergata, Via della Ricerca Scientifica, 00133 Rome, Italy; ilaria18scv@hotmail.it (I.B.); anastasia.deluca@uniroma2.it (A.D.L.); luisa.rossi@uniroma2.it (L.R.); 2 PhD School in Evolutionary Biology and Ecology, Department of Biology, University of Rome Tor Vergata, Via della Ricerca Scientifica, 00133 Rome, Italy; 3 Department of Chemical Science and Technologies, University of Rome “Tor Vergata”, 00133 Rome, Italy; fabio.domenici@uniroma2.it; 4 Instituto de Física, Universidad de Antioquia, Calle 70 No. 52-21, Medellín 050010, Colombia; cristiano.giordani@udea.edu.co; 5 Grupo Productos Naturales Marinos, Facultad de Ciencias Farmacéuticas y Alimentarias, Universidad de Antioquia, Calle 70 No. 52-21, Medellín 050010, Colombia

**Keywords:** antioxidant activity, breast cancer cells, glycophytes, *Lippia alba*, essential oil, phenolic compounds, ROS, salt stress

## Abstract

*Lippia alba* (Mill.) N.E.Br. ex Britton and P. Wilson is used in folk medicine of Central and South America for its biological activities: i.e., antifungal, antibacterial, antiviral, and anti-inflammatory. Based on ethnopharmacological information and the increasing interest in this species, this work aimed to test a possible wide use of its essential oil (EO) in pharmaceutical and horticultural applications. Therefore, we focused the attention on the antioxidant activity of the oil as a possible tool to overcome the oxidative stress in both applications. For this purpose, we have chosen three aggressive breast cancer cell lines and two horticultural species (*Solanum lycopersicum* L. and *Phaseolus acutifolius* L.) that are very sensitive to salt stress. We determined the antioxidant activity of *L. alba* EO through the quantification of phenols and flavonoids. Regarding tomato and bean plants under salt stress, *L. alba* EO was used for the first time as a seed priming agent to enhance plant salt tolerance. In this case, the seed treatment enhanced the content of phenolic compounds, reduced power and scavenger activity, and decreased membrane lipid peroxidation, thus mitigating the oxidative stress induced by salt. While in breast cancer cells the EO treatment showed different responses according to the cell lines, i.e., in SUM149 and MDA-MB-231 the EO decreased proliferation and increased antioxidant activity and lipid peroxidation, showing high cytotoxic effects associated with the release of lactate dehydrogenase, vice versa no effect was observed in MDA-MB-468. Such antioxidant activity opens a new perspective about this essential oil as a possible tool to counteract proliferation in some cancer cell lines and in horticulture as a seed priming agent to protect from oxidative damage in crops sensitive to salinity.

## 1. Introduction

Plants are a great source of nutraceuticals or phytochemicals, molecules with or without nutritional value but biologically active and beneficial for both plants and animals [1]. Natural products including crude extracts, essential oils, pure bioactive compounds extracted from plants, and herbal preparations have been shown to be the most common sources of phytochemicals [1]. The benefits of a diet rich in plants are based on the presence of various molecules, such as phytoestrogens, terpenoids, limonoids, phytosterols, carotenoids, and phenolic compounds, in particular flavonoids and anthocyanidins [2]. These compounds are widely distributed in plants, where they play key roles during plant development and interactions with the environment [1]. The antioxidant properties of some of these compounds have raised the interest of researchers [3]. Natural antioxidants, called free radical scavengers, may act as electron donors but are also able to break down peroxides and singlet oxygen or chelate metals [4]. Due to these characteristics, they play an important role against oxidative stress. The latter is one of the most studied biological conditions, due to many oxidative and antioxidative processes that can be observed under normal and pathological situations [4,5,6] in cells belonging to completely different organisms.

A typical hallmark of oxidative stress is the high intracellular levels of reactive species, molecules with low molecular weight and harmful for lipids, proteins, and DNA, but are also involved in redox signaling, providing proper physiological activities [7,8]. Reactive species can be divided into various groups. Among them, the most important are reactive oxygen species (ROS), but sulphur, nitrogen, halogen, and electrophilic reactive species can also be found [9]. In many biological systems, overproduction of ROS is a common consequence of stress; such overproduction can be detected in tumors, chronic inflammation, and bacterial and viral infections [10] in animal cells, while in plants the phenomenon is induced by either abiotic (e.g., salinity, drought, flooding) or biotic (e.g., insects, fungi infection) stresses [11,12].

Tumor cells have a higher level of ROS than normal cells due to the deregulation of their antioxidant systems [13,14]. In non-tumor cells, the antioxidant defense converts radical species into non-toxic molecules, but the onset of stress affects this balance, promoting the accumulation of radicals, particularly ROS. Oxidative stress is considered a key event in all stages of carcinogenesis (initiation, promotion, and progression of tumor) [1]: during initiation, ROS cause DNA damage by introducing mutations in tumor suppressor genes; in promotion phase, they inhibit apoptosis, maintaining a high rate of cell proliferation. During tumor progression, the persistence of oxidative stress contributes to the acceleration of mutagenesis in the uncontrolled proliferating cell population [1].

Among the tumors characterized by ROS overproduction, breast cancer is responsible for 23% of all cancer diagnoses in women worldwide and is the most important cause of death among female cancer patients [15,16]. Characterized by the lack of expression of estrogen receptor (ER-), progesterone receptor (PR-), and human epidermal growth factor receptor-2 (HER2-) [16], triple-negative breast cancer (TNBC) is the most aggressive one, and unfortunately, chemotherapy represents the only treatment against TNBC [15,16,17].

Although persistent oxidative stress condition can trigger carcinogenesis in animal cells, in plants it can lead to the death of the entire organism, unless antioxidant defense lines and tolerance responses are activated quickly [12]. Many abiotic stresses cause an overproduction of ROS that significantly damage plants, reducing their growth and yield. Soil salinization, due to a high amount of salt in the soil, is among the most dangerous abiotic stresses; it leads to an increase in the production of free radicals, in particular ^●^OH, ^●^O_2_^−^ and ^●^NO, resulting in an imbalance of cellular homeostasis [18]. Besides the damage to membrane proteins, lipids, and DNA, the ROS overproduction affects photosynthetic systems, metabolism, and inhibits water and ion uptake from the soil due to ionic and osmotic imbalances; high levels of soil salinity can lead to plant death [18,19].

**Figure 1:**
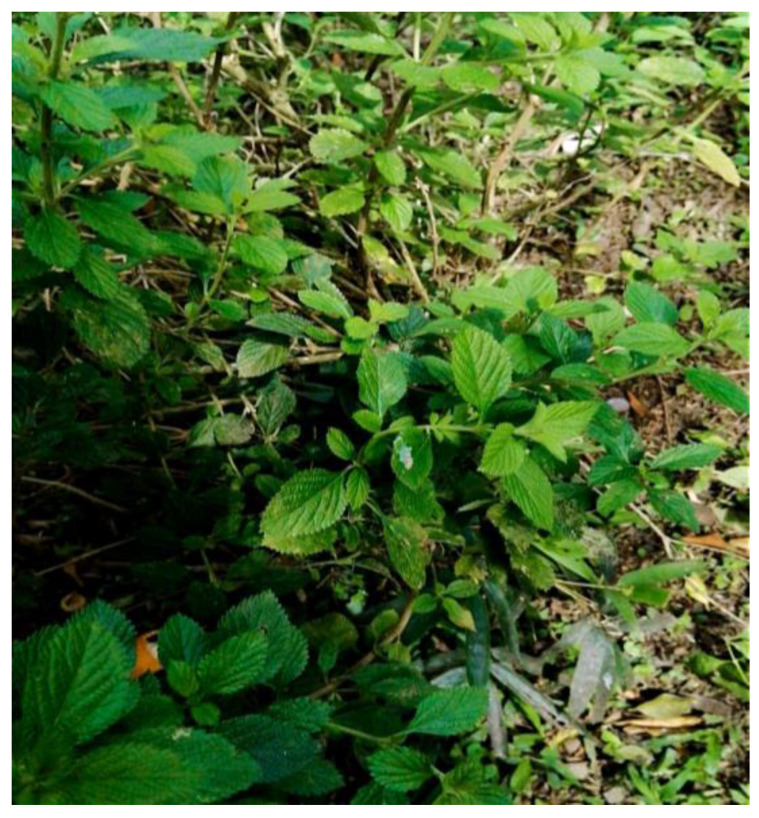
Habit of *Lippia alba* (Mill.) N.E.Br. ex Britton and P. Wilson.

The richness in antioxidant molecules in some plant species has raised the interest of researchers toward the use of them in both pharmaceutical and agronomic fields. Among the several plant species rich in phytochemicals and well-known to possess beneficial properties, we find *Lippia alba* (Mill.) N.E.Br. ex Britton and P. Wilson (Figure 1). Commonly known as ginger grass, it is an aromatic plant from South America used for the preparation of spices, infusions, and food integrators, as well as in traditional medicine and ethnopharmacological studies, thus arousing biotechnological interest [20,21]. According to Hennebelle et al. [22], infusions prepared with leaves and roots of *L. alba* are considered analgesic and sedative remedies and used to treat wounds, anemia, and skin diseases. Preparations based on *L. alba* are also used against digestive and respiratory disorders, and hypertension. The essential oil (EO) of ginger grass has a total of 93 compounds identified [22,23], but the major components are oxygenated monoterpenes (42.8%), monoterpene hydrocarbons (32.9%), and sesquiterpene hydrocarbons (21.9%), with carvone (35.2%), limonene (32.0%), and germacrene D (14.8%).

A wider utilization of EOs may open a new perspective in red and green biotechnologies. In particular, *Lippia* essential oil, rich in antioxidant compounds, may play an important role in counteracting the negative effects of ROS on both animal and plant cells. Thus, the aim of this work was to verify if ginger grass EO has the potential to counteract oxidative stress in two completely different cells, where the level of ROS is high, e.g., tumor cells and plant cells exposed to stress conditions. For this purpose, we tested the effects of EO of *L. alba*, belonging to the limonene/carvone chemotype [23]. Limonene and carvone possess beneficial properties; carvone has decongestant, diuretic, and antiviral properties, while limonene prevents bronchitis, diabetes, gallstones, heartburn from gastroesophageal reflux, cholesterol, and cancer processes [24].

The experiments were undertaken on very aggressive triple-negative breast cancer cell lines and on two plant species very sensitive to saline conditions: i.e., *Phaseolus acutifolius* L. (from the same geographical area of *L. alba*) and *Solanum lycopersicum* L. (an important crop, rich in anticancer compounds and phytochemicals), both exposed to salt stress. The single treatment is reported and described in Materials and Methods.

## 2. Results

### 2.1. Quantification of Phenolic Compounds and Antioxidant Activity of EO

**Figure 2:**
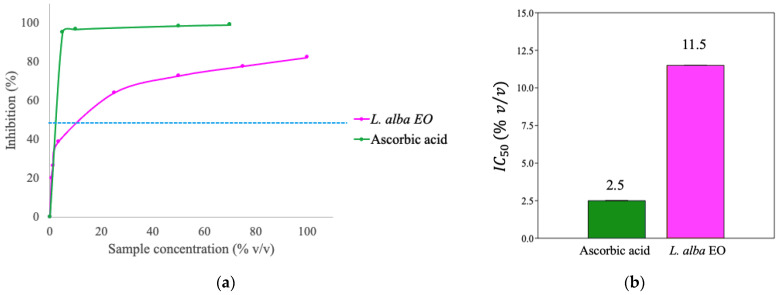
DPPH free radical inhibiting activity (%) at various concentration of EO (% *v*/*v*) (**a**) and estimation of IC_50_ value of EO (**b**). The dotted line represents 50% inhibition. The values are compared with ascorbic acid.

Phenolic compounds, in particular, flavonoids, the most common class of antioxidant secondary metabolites, were detected in the EO (Table 1). Moreover, the antioxidant power of EO was evaluated by DPPH free radical assay (Figure 2a), and IC_50_ value was calculated and compared with that of ascorbic acid, used in the test as positive control (Figure 2b). The IC_50_ of the oil was 4.6 times lower compared to the IC_50_ of ascorbic acid (Figure 2b), the most powerful antioxidant molecule.

### 2.2. Effect of EO in Plants 

The use of EO led to a significant improvement in the growth of both species (Supplementary Materials Figures S1 and S2). The increase in soil salinity, verified by the analysis of electrical conductivity (EC), decreased the shoot length of all bean plants (primed and not primed) (Table 2). However, the shoots and biomass of stressed primed beans were significantly higher compared to not primed and stressed beans plants (Table 2). Damage caused by salinity (reduction of shoots and biomass) was also observed in primed and not primed tomato plants (Table 2); nevertheless, primed plants showed better parameters when compared to not primed ones grown under the same condition (Table 2). These data indicate that the EO seed priming, followed by acclimation, led to a significant improvement of growth in saline conditions (Table 2).

### 2.3. Effects of EO on Secondary Metabolites 

**Figure 3:**
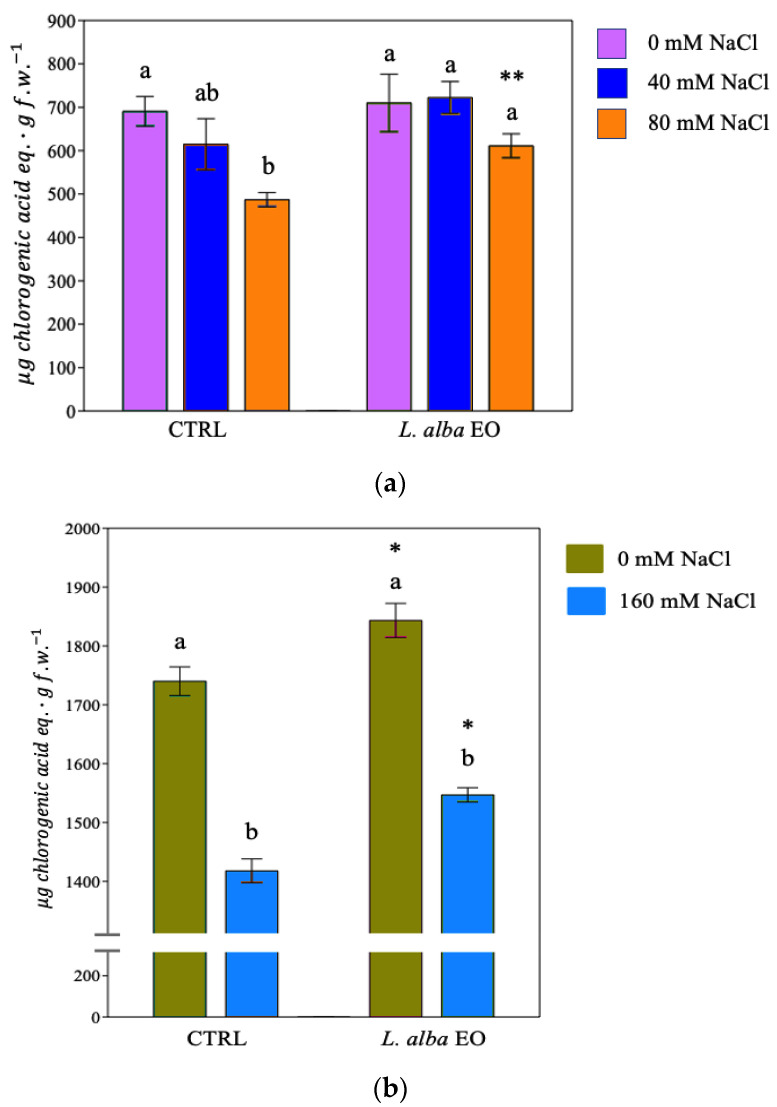
Phenolic compounds of bean (**a**) and tomato (**b**) plants. Data are expressed as mean ± SE (*n* = 6). Mean values in the column marked by different letters are significantly different within the same group (*p* < 0.05; ANOVA and Tukey–Kramer test). Significant differences to CTRL are reported as * *p* < 0.05; ** *p* < 0.01.

**Figure 4:**
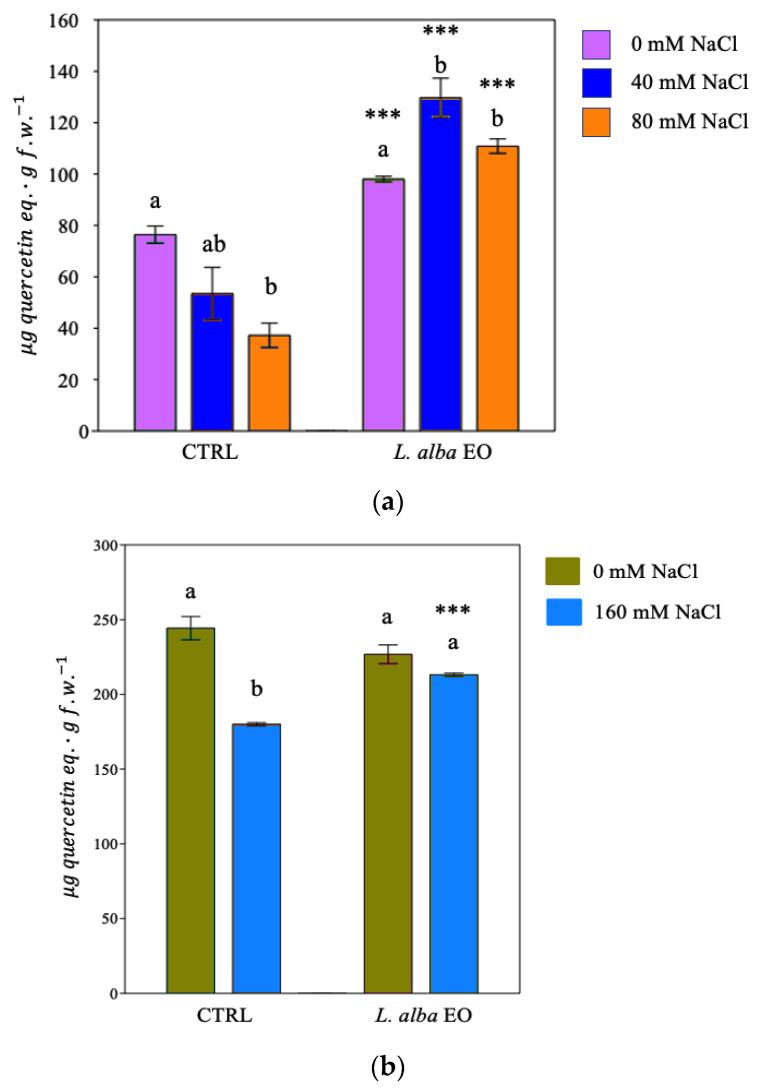
Flavonoids of bean (**a**) and tomato (**b**) plants. Data are expressed as mean ± SE (*n* = 6). Mean values in the column marked by different letters are significantly different within the same group (*p* < 0.05; ANOVA and Tukey–Kramer test). Significant differences to CTRL are reported as *** *p* < 0.001.

Salt irrigation led to a reduction in the synthesis of phenols and flavonoids in control plants (CTRL) of both crops (Figure 3a,b and Figure 4a,b).

The concentration of phenols in bean plants decreased by 30% (Figure 3a) and flavonoids by 50% following the irrigation with 80 mM NaCl (Figure 4a). While no significant reduction in the amount of the phenol was observed in primed plants (Figure 3a), the amount was significantly higher in stressed primed plants compared to the relatively not primed controls. A significant enhancement of flavonoids was found in all primed plants with respect to not primed controls (Figure 4a).

A similar pattern was detected in tomatoes, where the priming significantly increased the amount of phenols (+8.4%) and flavonoids (+37.5%) compared to stressed and not primed controls (Figure 3b and Figure 4b).

### 2.4. Total Antioxidant, Reducing Power, and Scavenger Activity in Plants 

**Figure 5:**
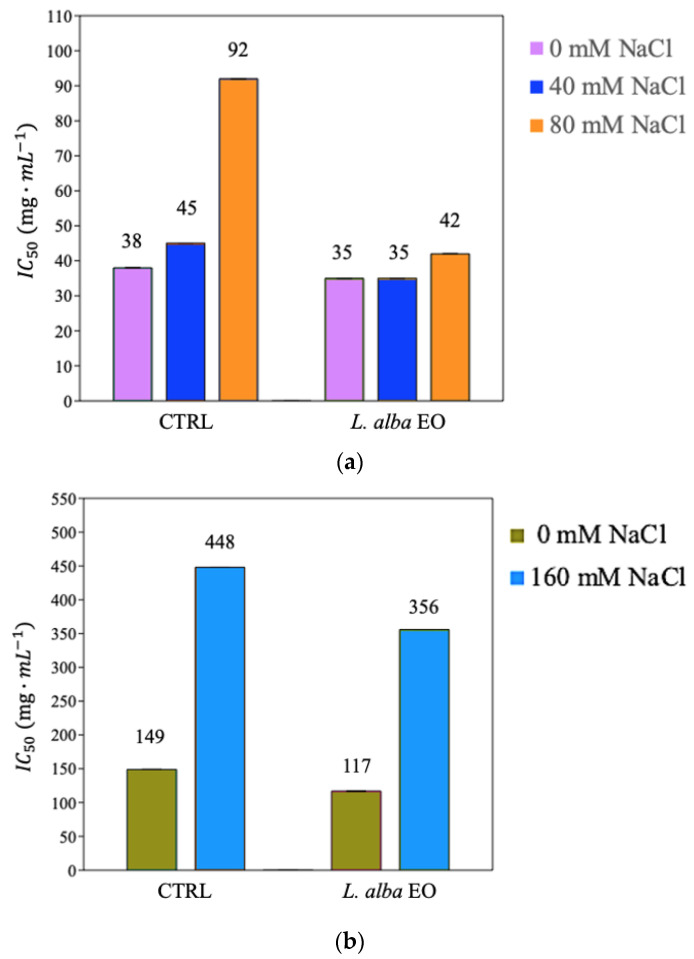
Estimation of IC_50_ value of bean (**a**) and tomato plants (**b**) expressed as mg·mL^−1^.

Antioxidant activity, reducing power, and scavenger activity of the plants were analyzed by the DPPH, FRAP, and P-FRAP tests, respectively. Salinity decreased the antioxidant activity of not primed bean and tomato plants (Figure 5a,b, Figure S3a–c and Figure S4a,b). On the contrary, EO improved the antioxidant activity, reducing power and scavenger activity of all stressed and not stressed primed plants compared to not primed CTRLs (Figure 5a,b, Figure S3a–c and Figure S4a,b, Table 3). In particular, the antioxidant activity of bean primed plants irrigated with 80 mM NaCl (Figure 5a) showed the IC_50_ of 42 mg/mL vs. 92 mg/mL of CTRL. Similar behavior was observed in tomatoes, leading to a better salinity tolerance (Figure 5b).

### 2.5. Membrane Lipid Peroxidation Inhibition in Plants 

**Figure 6:**
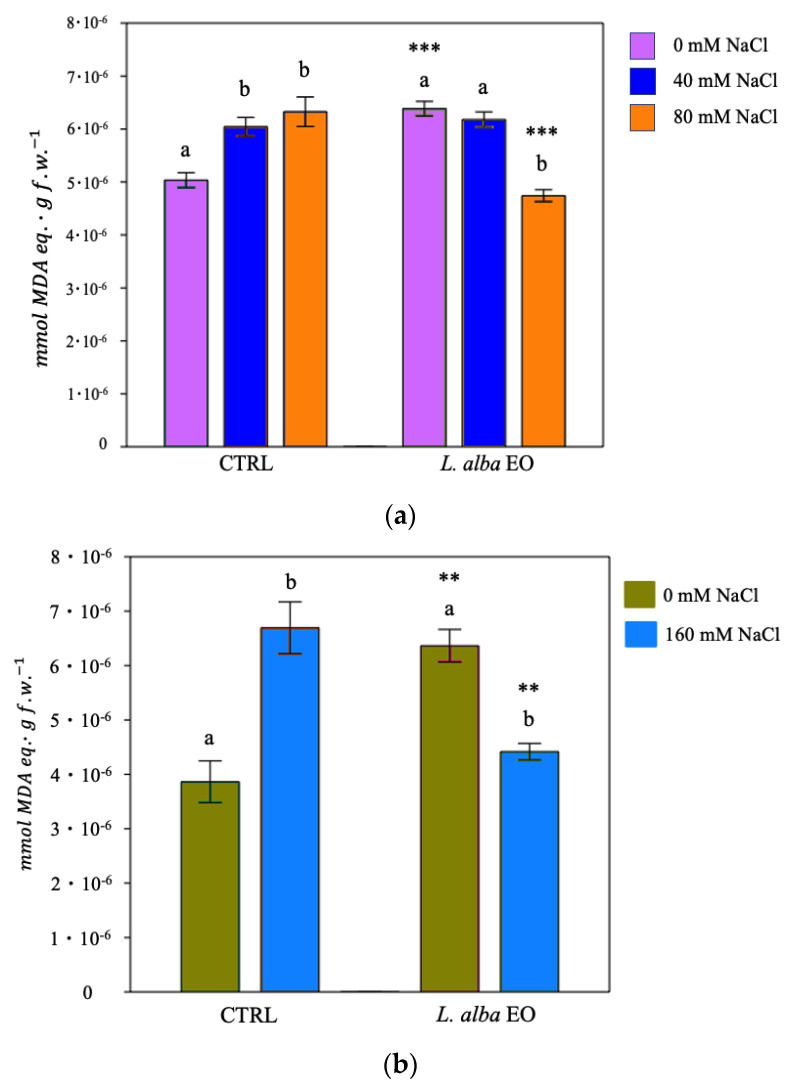
Thiobarbituric acid reactive products in bean (**a**) and tomato (**b**) samples. Data are expressed as mean ± SE (*n* = 6). Mean values in the column marked by different letters are significantly different within the same group (*p* < 0.05; ANOVA and Tukey–Kramer test). Significant differences to CTRL are reported as ** *p* < 0.01; *** *p* < 0.001.

The study of thiobarbituric acid (TBA) reactive products was made possible to determine the damage caused by peroxidation of the plasma membrane lipids. High malondialdehyde (MDA) level, expressed as mmol malondialdehyde (MDA) eq./g f.w., indicates a high level of lipid peroxidation. In the control plants of both species, an increase in salinity corresponded to a higher level of lipid peroxidation, whereas seed priming showed a protective effect, by decreasing the amount of MDA under stress conditions in both species (Figure 6a,b), in particular, −23% and −34%, respectively, in bean and tomato at high levels of salinity irrigation.

### 2.6. Effect of EO on Proliferation and Cytotoxicity of MB-231, SUM149 and MB-468

**Figure 7:**
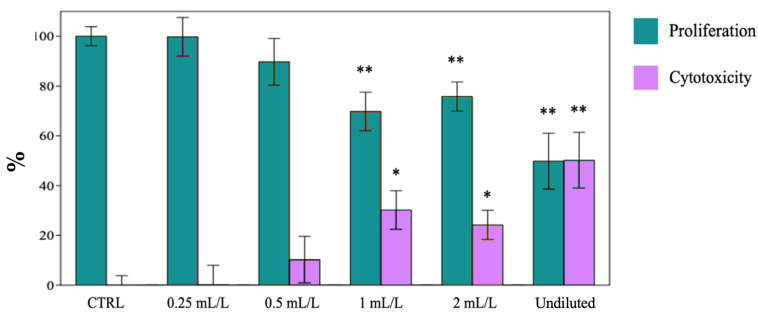
Proliferation and Cytotoxicity (%) of MDA-MB-231 cell lines. Data are expressed as mean ± SE (*n* = 6). Significant differences to control (CTRL) were calculated by *t*-student test and reported as * *p* < 0.05; ** *p* < 0.01.

**Figure 8:**
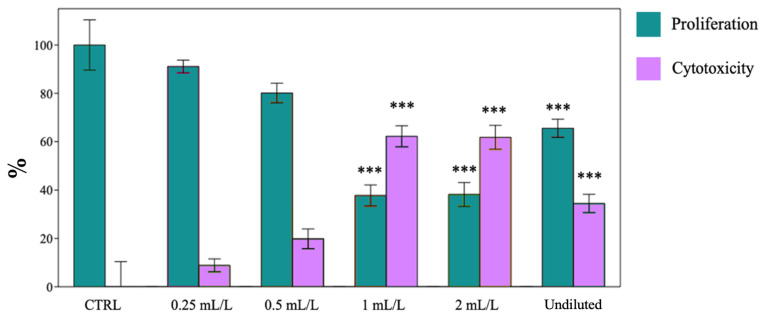
Proliferation and Cytotoxicity (%) of SUM149 cell lines. Data are expressed as mean ± SE (*n* = 6). Significant differences to control (CTRL) were calculated by *t*-student test and reported as *** *p* < 0.001.

The effect of EO on cancer cell proliferation and its cytotoxicity was concentration-dependent, even though showing important differences among the three cell lines was considered. Increasing concentrations of EO significantly reduced the proliferation of MDA-MB-231 cells with increasing toxicity when compared to the control cells (CTRL) treated with DMSO (Figure 7). The most significant effects on cell viability were observed in SUM149, where 1 and 2 mL·L^−1^ of EO led to 70% of cell death (Figure 8). The peculiar effects, observed in SUM149 treated with undiluted EO, could be a consequence of the lower solubilization of oil in the culture medium, which may have reduced the antiproliferative and cytotoxic potential of the EO, leading to the results reported in Figure 8.

**Figure 9:**
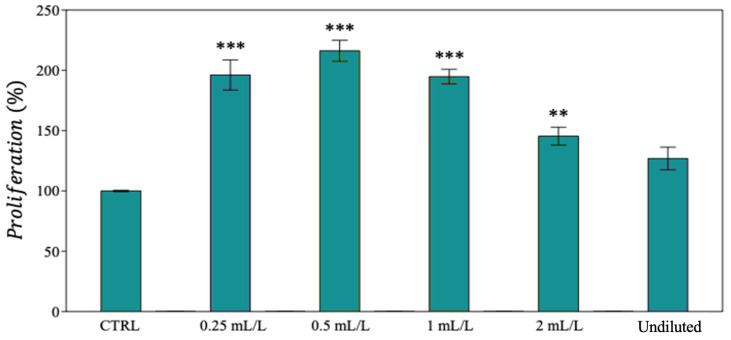
Proliferation (%) MDA-MB-468 cell lines. Data are expressed as mean ± SE (*n* = 6). Significant differences to control (CTRL) were calculated by *t*-student test and reported as ** *p* < 0.01; *** *p* < 0.001.

Due to the limited solubilization of undiluted EO in the culture medium, at the end of this first set of experiments, it was decided to treat the MDA-MB-231 and SUM149 cell lines with oil at C_f_ = 2 mL·L^−1^. An opposite scenario was observed in MDA-MB-468 after 48 h of treatment: i.e., an increase of proliferation (from +25% to +120%) was detected in the treated cells (Figure 9). In the latter, the treatment seems to act as an activator of cell proliferation, in contrast to what was observed in the other cell lines, where the oil exhibited a strong cytotoxic effect. This was the reason why MDA-MB-468 cells were excluded in the following tests.

### 2.7. Total Antioxidant, Reducing Power, and Scavenger Activity of MB-231 and SUM149 Treated with EO

**Figure 10:**
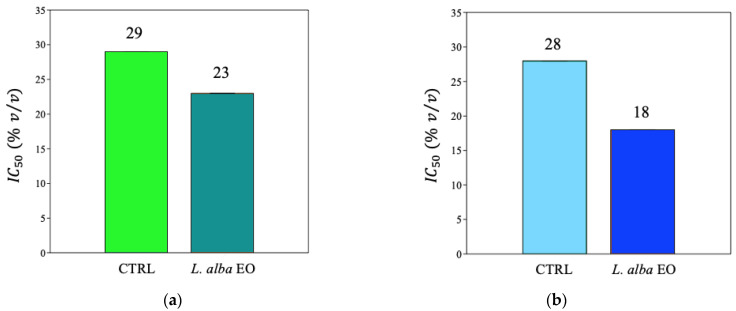
Estimation of IC_50_ value from MDA-MB-231 (**a**) and SUM149 (**b**) cell lines expressed as % *v*/*v*.

**Figure 11:**
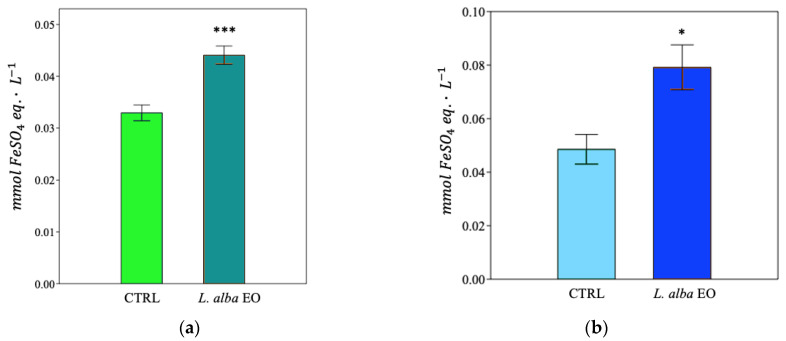
Ferric reducing antioxidant power of MDA-MB-231 (**a**) and SUM149 (**b**) cell lines. Data are expressed as mean ± SE (*n* = 6). Significant differences to control (CTRL) were calculated by *t*-student test and reported as * *p* < 0.05; *** *p* < 0.001.

**Figure 12:**
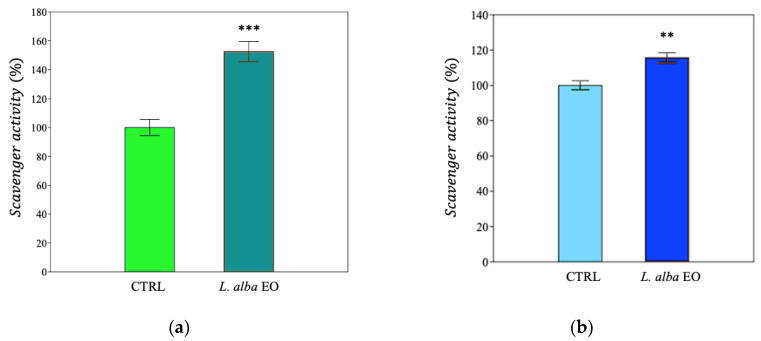
Potassium ferricyanide reducing antioxidant power of MDA-MB-231 (**a**) and SUM149 (**b**) cell lines. Data are expressed as mean ± SE (*n* = 6). Significant differences to control (CTRL) were calculated by *t*-student test and reported as ** *p* < 0.01; *** *p* < 0.001.

In the MDA-MB-231 cell line, upon treatment with EO, we determined an increase in antioxidant activity, as IC_50_, (Figure 10a and Figure S5a), as well as a significant improvement in reducing power and scavenger activity, i.e., +31.3% and +60%, respectively, when compared to the CTRL (Figure 11a and Figure 12a). A similar behavior was detected in SUM149, where the antioxidant activity improved in samples treated with oil (Figure 10b and Figure S5b), with increments of +60% and +20%, respectively, in the ferric and potassium ferricyanide reducing power and scavenger activity, found in treated samples when compared to CTRL (Figure 11b and Figure 12b).

### 2.8. Markers of Cell Damage: Thiobarbituric Acid (TBA) Reactive Products and Lactate Dehydrogenase Activity (LDH) 

**Figure 13:**
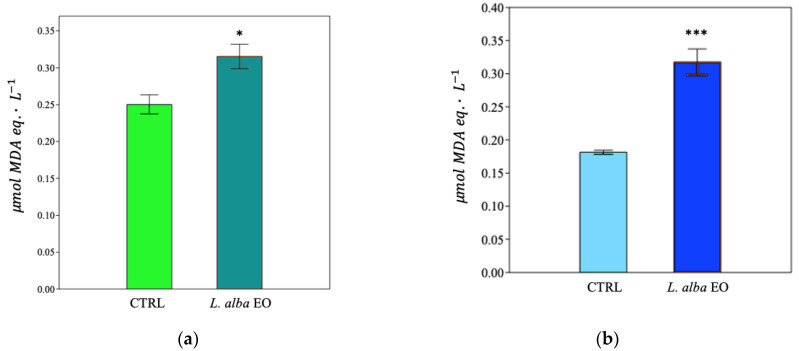
Thiobarbituric acid reactive products of MDA-MB-231 (**a**) and SUM149 (**b**) cell lines. Data are expressed as mean ± SE (*n* = 6). Significant differences to control (CTRL) were calculated by *t*-student test and reported as * *p* < 0.05; *** *p* < 0.001.

The quantification of the reactive products of TBA made it possible to estimate the degree of membrane lipid peroxidation, expressed as μmol of malondialdehyde (MDA) equivalent·L^−1^. EO treatment increased lipid peroxidation damage in both MDA-MB-231 and SUM149 by +24% and +88%, respectively, compared to CTRL (Figure 13a,b).

**Figure 14:**
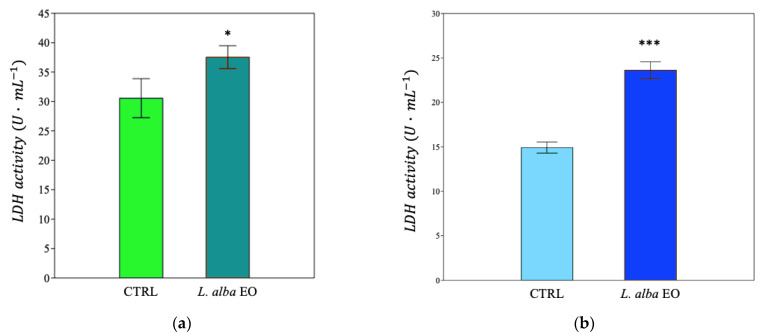
Lactate dehydrogenase activity of MDA-MB-231 (**a**) and SUM149 (**b**) cell lines. Data are expressed as mean ± SE (*n* = 6). Significant differences to control (CTRL) were calculated by *t*-student test and reported as * *p* < 0.05; *** *p* < 0.001.

The toxic effect of EO on cell lines was further investigated by measuring the activity of the LDH enzyme, released from the damaged cells in the culture medium. LDH found in the cytoplasm catalyzes the conversion of lactate into pyruvate, reducing NAD^+^ into NADH. The more LDH is released in the culture medium, the higher is the cell damage [25]. Significant enhancement of LDH activity was observed in both cell lines (+23.3% and +53.3%, respectively, in MDA-MB-231 and in SUM149, compared to relative CTRL) (Figure 14a,b).

## 3. Discussion

Plants are a huge source of numerous valuable natural products. These compounds, called phytochemicals, play multiple roles in plant-environment interaction, providing plant protection against diseases, pests, and abiotic stresses. Some of these molecules are also considered important drugs for pharmaceutical therapy. Among plant-based products, the antioxidants are considered one of the most promising and important molecules for their biological activity. Plant essential oils share complex composition, most of the time including antioxidant bioactive compounds, such as terpenoids and phenylpropanoids [26]. In folk medicine, worldwide, EOs have been used empirically in the treatment of different diseases; researchers have characterized several biological properties of EOs, among them antimicrobial, antiviral, antimutagenic, anticancer, antioxidant, anti-inflammatory, immunomodulatory, and antiprotozoal activities [27]. Obviously, their effects on living organisms are depending on the concentrations required to affect either the growth or the metabolisms of the target organisms [26]. Among the several EOs available on the market, *L. alba* oil is used in traditional medicine and it is the core of ethnopharmacological studies [20,21]. According to the researches performed by Stashenko et al. [22] and Benelli et al. [23], the components of *Lippia* EO showed antioxidant, antibacterial, and antifungal activities. Data on antitumor activity are also reported in the literature [28]. 

Basing on the characteristics of the components, *L. alba* EO may have different applications in human health and agriculture. The determination of antioxidant properties of the oil of *L. alba* was performed by analyzing the content of phenols and flavonoids, which concentrations are 177.3 ± 4.2 μg of chlorogenic acid equivalents·mL^−1^ and 17.2 ± 2.1 μg of quercetin equivalents·mL^−1^, respectively. The presence of these secondary metabolites in the EO allowed the evaluation of the antioxidant power using the DPPH assay and the calculation of the IC_50_. This value was then compared with the IC_50_ of ascorbic acid. Even thought, the antioxidant power of *L. alba* oil was found to be 4.6 times lower than ascorbic acid, this value was higher with respect to other plant extracts reported in the literature [29,30]. After the determination of the presence of antioxidant compounds in oil, the study focused on the effects of EO treatment in tumor cells and plants exposed to stress conditions.

### 3.1. Mechanism of Action of EO in Salt-Stressed Plants

Seed priming with different compounds is a technique used in green biotechnology to improve stress response of the plants; under this perspective, EOs obtained from medical and aromatic plants can be a useful priming agent [31]. For example, phenols, flavonoids, tannins, alkaloids, saponins, and sterols found in EOs are a valid priming agent, alternative to the chemical compounds currently used (e.g., NaCl, Ca(NO_3_)_2_, MgSO_4_, gibberellins, etc.) [31].

Although there are scientific papers concerning the biotechnological use of EOs as priming agents [32,33], there are no literature studies regarding the mechanism of action of *L. alba* EO in plants exposed to salt stress. The latter is responsible for the onset of ionic, osmotic, and oxidative stress in plants, leading to the death of the organism if enzymatic and non-enzymatic antioxidant defence systems are not quickly activated [34]. ROS production is the most damaging and fatal event for a plant under stress conditions; such toxic molecules damage DNA, proteins, plasma membrane, and cytoplasmic organelles, when the antioxidant mechanism are absent or not quickly activated [35].

Some secondary metabolites are components of the non-enzymatic antioxidant system, i.e., phenolic compounds, particularly, flavonoids that are widely distributed in the plant kingdom [34,36]. These compounds play various roles in plants: they are considered signaling and defense molecules, providing a significant contribution to free radical scavenging [36]. As shown by many studies, the environmental saline condition activates the signaling pathways for the synthesis of secondary metabolites. This process, present in halophyte species [37], does not occur in glycophytes, such as tomato and bean, where an increase in salinity corresponds to a decrease in phenols and flavonoids production [12,38].

The use of *L. alba* EO as a priming agent allowed the improvement of the synthesis of phenolic compounds, thus reducing the oxidative stress caused by prolonged irrigation with saline solutions, and in the meantime it increased salt tolerance. Differently from what was reported by Atak et al. [32], who describe a negative effect of EOs (*Origanum onites* L. and *Rosmarinus officinalis* L.) on plants, in our experiments the higher production of phenolic compounds led the primed plants to enhance their antioxidant and scavenger activity, and reducing power, data confirmed by IC_50_ analysis and also by FRAP and P-FRAP assays. The different response may be due to the allelopathic effect of the EOs [32], but also to the chemotype of the plant, responsible for active principles present in EO, beside the species-specific properties of the plant and even the cultivar chosen for the study.

Flavonoids are able to inhibit lipid peroxidation [39], which is defined as the mechanism where molecules, like ROS, attack lipids of the plasma membrane, in particular polyunsaturated fatty acids (PUFAs) rich in carbon-carbon double bonds, generating other radical species (e.g., lipid peroxyls and hydroperoxides) [40]. Indeed, salt stress is known to induce extensive lipid peroxidation, resulting in an accumulation of malondialdehyde, which is considered a good marker of salt-induced oxidative stress damage in plasma membranes [41]. The accumulation of ROS, induced by salinity, causes lipid oxidation, thus affecting the composition of the plasma membrane, and consequently their ultrastructure, decreasing the fluidity and modifying the permeability [12,38]. Our data showed a decrease of MDA content and a reduction of oxidative damage, suggesting protective action of EO on membrane lipids.

**Figure 15:**
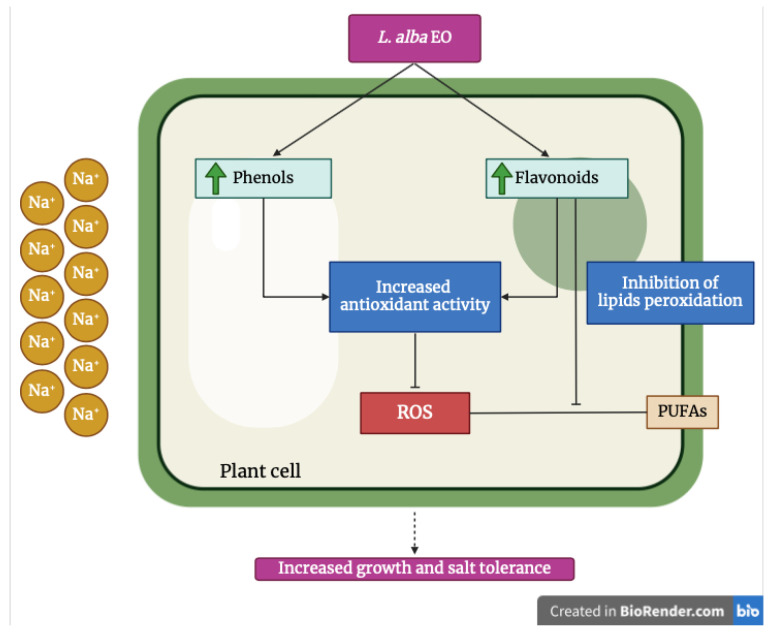
Possible mechanism of action of *L. alba* EO on bean and tomato cells under salt stress. In the presence of high salt concentration, primed plants increase the synthesis of intracellular phenolic compounds, enhancing their antioxidant activity, inhibiting ROS overproduction. Furthermore, flavonoids prevent lipid peroxidation, avoiding the interaction between ROS and PUFAs, which cause severe damage to the plasma membrane by producing new radical species. Created by BioRender.com (accessed on 23 July 2023).

Based on our results, we can conclude that the EO of ginger grass, unlike from other oils previously used in different horticultural crops, is a good priming agent of both crop species tested, i.e., EO priming reduced oxidative damage caused by prolonged exposure to salt. The protective action of the oil improved significantly the growth of both crops under stress conditions, making them more tolerant to salinity. A possible mechanism of action of EO of *L. alba* is reported in Figure 15.

### 3.2. Mechanism of Action of EO in Breast Cancer Cells

Since cancer is the second-highest cause of death worldwide, the studies on the mechanisms underlying tumor progression led to the development of numerous anti-cancer drugs. However, in many patients, the use of chemotherapy drugs has collateral effects, like fatigue, hair loss, anemia, nausea and vomiting, peripheral neuropathy, weight changes, and fertility problems [42]. Therefore, natural plant compounds can represent key resources for the development of new cancer drugs and therapies [43], reducing adverse reactions. Phytochemicals are involved in molecular pathways of cancer growth and progression, inactivating carcinogens, inhibiting proliferation, inducing cell cycle arrest, and apoptosis [44].

The cytotoxic and antitumor effects of *L. alba,* and in particular of some of the major components of essential oil, i.e., limonene and citral, have been demonstrated in HL-60 (human promyelocytic leukaemia cells), K562 (human erythroleukemic cells), HepG2 (human hepatocellular carcinoma cells), and HeLa (human cervix epithelioid carcinoma cells) [45]. However, controversy exists in literature, since the extracts of carvone and citral chemotypes of *L. alba* showed low cytotoxicity on HeLa cells. One of the principal components of EO, i.e., carvone, has been reported to possess antioxidant, antimicrobial, and antitumor activities: i.e., in cultured primary rat neuron cells, the molecule increased levels of total antioxidant capacity [22,23,46,47].

The antitumor effect of *L. alba* has already been reported in previous research [48,49,50], where the capacity of extract to inhibit tumor proliferation and promote cell differentiation was highlighted. In our study, even though *L. alba* EO significantly reduced cell growth, the results were different according to the tumor line. Whereas the oil reduced proliferation in MDA-MB-23 and even more in SUM149, a totally opposite effect was observed in MDA-MB-468, showing a marked increase in proliferation when treated with the oil. Such differences could be due to specific genetic factors, intrinsic resistance of MDA-MB-468, alterations in membrane transporters, or cell metabolism [51,52], which makes these cells chemoresistant. These results led to the decision of excluding the latter cell line from the following analyses.

In both SUM149 and MDA-MB-231, the threshold value of cytotoxicity was found to be 1 mL·L^−1^; EO concentrations below this value were not toxic for the cells. However, since it is an essential oil and not a purified molecule, it was decided to treat SUM149 and MDA-MB-231 with an EO solution of 2 mL·L^−1^ to increase the cytotoxicity of the oil. Since the toxicity of many anticancer agents often involves the generation of ROS and alteration of redox state, which both are commonly biochemical changes observed in cancer cells [5,53,54], in our experiments the cytotoxicity tests were followed by biochemical analyses on the antioxidant activity of EO. Cells can tolerate a certain level of oxidative stress due to their antioxidant capacity, which prevents transformation and death [10]. An increase in ROS can inhibit tumor cell growth; in advanced tumors, a further increase in oxidative stress, due to the use of chemotherapy or radiotherapy, can overcome the cells’ antioxidant defenses, leading to cell death [5]. In cancer cells, elevated ROS generation is due to metabolic anomalies, and oncogenic signaling activates an adaptive response, resulting in an up-regulation of antioxidant activity to keep ROS levels below the toxic threshold [13]. Although the enhancement of antioxidants promotes resistance to treatment, the use of this mechanism to prevent the blocking of mitosis, the induction of senescence and death, is a double-edged sword for tumor cells, having pro-tumorigenic or cytotoxic effects, depending on the concentration [14].

A further increase in oxidative stress, caused by exogenous agents that modulate ROS or lipid peroxidation substrates, leads to an excess of ROS above the cytotoxic threshold, resulting in tumor cell death [5,7,13]. Therefore, tumor cells use enhanced antioxidant defense systems to reduce the accumulation of free radicals [7,13,14]. Our data confirm a similar mechanism of response [7,14] in both lines treated with *L. alba* oil: a significant increase in antioxidant, reducing, and scavenger power was observed. The up-regulation of antioxidant power, induced by EO, indicates that there was an overproduction of ROS in cells, which exceeded the cytotoxicity threshold. Although the cells tried to balance this overproduction, this was not sufficient to cope with the toxicity level which would allow their survival. Overcoming the threshold toxicity limit induced cell cycle arrest and death, supporting the previous observations regarding proliferation and cytotoxicity. The interaction between the unstable ROS and PUFAs of membranes induces the peroxidation of the lipids, which products are considered second messengers of oxidative stress [55,56,57,58]. It follows that cell membrane is particularly vulnerable to ROS damage due to high levels of PUFA. Cancer cells are more sensitive than normal cells to the accumulation of ROS, so the incorporation or enrichment of PUFA may lead to deeper negative effect on lipid peroxidation. PUFAs and anticancer agents have been shown to produce a synergistic cytotoxic effect in various tumors; in all these studies, cytotoxicity was mediated by an increase of lipid peroxidation products, like MDA [54,59]. This evidence suggests that lipid peroxidation products could provide a therapeutic tool to induce the death of proliferating tumor cells, enhancing the toxicity of anticancer agents and radiotherapy.

**Figure 16:**
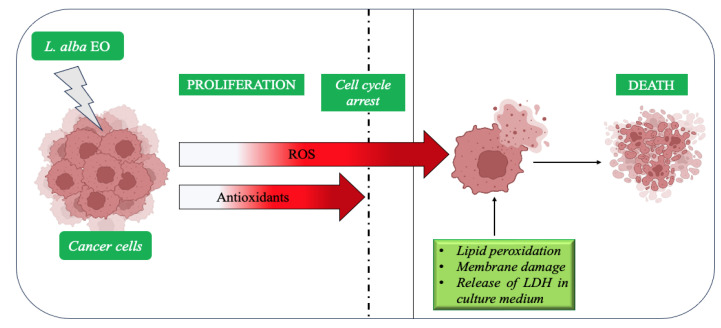
Possible mechanism of action of *L. alba* EO on breast cancer cells. The dotted line represents the threshold value of antioxidant capacity of the cells to overcome ROS toxicity. Above this threshold, the cells are unable to overcome the damage caused by the accumulation of ROS. (Image based and modified from [5,14]). Created in BioRender.com (accessed on 23 July 2023).

According to [57,59,60], the treatment of the cancer cells with *L. alba* oil produced a marked increase in lipid peroxidation, detected as an enhancement of MDA concentration. The latter indicated a marked level of oxidative stress in the treated lines compared to the controls. The elevated value also confirmed previous data on proliferation and cytotoxicity tests: treated cells showed a lower growth rate, probably due to an increase in ROS concentration. The latter was related to the production of lipid peroxyl radicals and hydroperoxides, uncontrolled lipid peroxidation leading to membrane disruption. The loss of membrane integrity results in the release of LDH into the extracellular space, an enzyme, which activity can be used as a biomarker of cell viability [61,62]. The release of intracellular enzymes through the damaged cell membrane can be a consequence of alteration of the mitochondrial machinery and deregulation of apoptosis. These data are a further confirmation to all previous observations concerning the toxicity of EO and agree with the data of the literature concerning LDH [61,62]. The treatment with *L. alba* EO seems to act as an activator of cell death, based on the loss of membrane integrity, resulting in the release of cellular content into the surrounding environment. A hypothesis of possible mechanism of action of EO of ginger grass is reported in Figure 16.

## 4. Materials and Methods

During experiments, all working solutions were freshly prepared from stock reagents. 

The EO of *L. alba* was purchased from Centro de Investigación de Excelencia—CENIVAM (http://quim.uis.edu.co/eisi/grupo/cenivam/#views/gm1/inicio (accessed on 15 May 2024)), Bucaramanga, Santander, Colombia. The EO was obtained from fresh leaves and flowers of *L. alba* plants; EO extraction was performed by using the microwave-assisted hydrodistillation method, as described by Stashenko et al. [22].

The oil was stored in glass bottles at −20 °C in the dark and stabilized at room temperature (RT) just before each test. The composition of EO has been described by Benelli et al. [23]. To determine if *L. alba* EO contained other secondary metabolites with antioxidant activity, preliminary tests were conducted using undiluted and diluted EO. Two different experimental set-ups were used for plant and cell growth, as reported below. 

### 4.1. Plants’ Growth Conditions and Experimental Setting

Seeds of *Solanum lycopersicum* L., cv. Principe Borghese, were used and purchased from Blumen Group S.p.A (Piacenza, Italy), while seeds of *Phaseolus acutifolius* L., cv. Blue Tepary, were chosen and purchased from a specialized store near the University of Rome Tor Vergata. Seeds were stored in the dark at RT and, before priming, they were surface sterilized in 70% ethanol for 5 min, immersed in a solution of 1% NaClO for 5 min, and rinsed in double distilled water.

Both tomato and bean were very sensitive to salt stress. Therefore, based on previous experiments [12,38], we adopted two strategies to increase plant tolerance to salt, such as acclimation and seed priming [12,38].

To determine the optimum concentration of EO for priming treatment, preliminary tests were carried out with EO at various concentrations: 0 mL·L^−1^, 0.25 mL·L^−1^, 0.5 mL·L^−1^, 1 mL·L^−1^, and 2 mL·L^−1^. Based on the germination (%) of the seeds, 0.5 mL·L^−1^ was detected as the best priming solution. We primed the seeds in 50 mL of 0.5 mL·L^−1^ of EO for 24 h at RT. At the end of the treatments, seeds were rinsed with double distilled water and air dried at RT for 48 h.

The seeds were germinated in Petri dishes (10 seeds each), soaked in 10–15 mL of double distilled water, and incubated in the dark for 10 days at RT. Four germinated seeds were sown in plastic pots (15 cm diameter), containing about 300 g of soil (COMPO SANA^®^ COMPACT, Münster, Germany). Soil characteristics were already reported in [12,38]. The plants were grown in a greenhouse under natural sunlight (Daily Light Integral: 89 mmol/m^2^ day ± 16 mmol/m^2^ day) at a temperature of 23 °C ± 2 °C. Growth conditions were monitored every day with a multi-parameter sensor (Flower Care—HHCCJCY01HHCC—HHCC Plant Technology Co., Ltd.—Stuttgart, Germany).

Bean and tomato seedlings were grown for 14 days before the beginning of salt treatments. Salt concentrations of irrigation water were chosen according to previous experiments, where such concentrations were the threshold of salt tolerance of each species [12,38]. Bean saline water consisted of the following solutions: 40 mM NaCl (EC: 4.2 dS/m) and 80 mM NaCl (EC: 8.5 dS/m). Tomato saline water consisted of the solution of 160 mM NaCl (EC: 15.9 dS/m).

For both species the pots were randomly assigned to the experimental sets: (1) not primed seeds (control, CTRL) irrigated with tap water (EC: 0.5 dS/m) or with saline solutions (control stressed); (2) primed seeds irrigated with tap water or with saline solutions. Primed and not primed bean plants were watered with 100 mL of tap water or salt solution every 48 h for 4 weeks. Primed and not primed tomato plants were watered with 80 mL of tap water or salt solution every 72 h for 4 weeks. At the end of the experimental periods, morphological parameters were analyzed and the electrical conductivity (EC) of the soil was detected according to Santangeli et al. [11] using an EC meter (HANNA Instrument 98312 DiST^®^5 and DiST^®^6, Padova, Italy). Plants were sampled, frozen by dipping in liquid nitrogen, and stored at −20 °C until further analyses.

### 4.2. Plants Samples Preparations 

The preparation of samples for the quantification of phenolic compounds required a preliminary step of homogenization of 0.2 g of frozen material, performed according to Borromeo et al. [12].

The preparations of samples for antioxidant assays (DPPH, FRAP, and P-FRAP) and thiobarbituric acid reactive products (TBARS) assay were carried out by grinding 0.2 g of the frozen material respectively in 5 mL of 100% methanol (Merck KGaA, Darmstadt, Germany) and in 1 mL of double distilled water. The samples were left in the dark at +4 °C overnight, centrifuged at 8000× *g* for 15 min, and the supernatants were collected and stored at −20 °C until further analysis.

### 4.3. Determination of Phenolic Compounds in EO and in Plant Cells 

The quantification of phenols and flavonoids involved both plant samples and EO. The total phenolic content was quantified according to Santangeli et al. [11], using Folin–Ciocalteau reagent (Merck KGaA). The absorbance was measured at 724 nm with a spectrophotometer (VARIAN Cary 50 Bio, Santa Clara, CA, USA). The concentration of phenols was evaluated using a calibration curve of chlorogenic acid (y = 0.004x + 0.0094; R^2^ = 0.999) (Merck KgaA) and expressed as μg chlorogenic acid equivalent·mL^−1^ and μg chlorogenic acid equivalent·g f.w.^−1^ (for EO and plant material, respectively).

Flavonoids were quantified according to Chang et al. [63] with modifications reported in [12]. The absorbance was measured at 415 nm with a spectrophotometer (VARIAN Cary 50 Bio) and calculated using a calibration curve of quercetin (Merck KgaA) as standard (y = 0.0068x + 0.0271; R^2^ = 0.998). Flavonoids were expressed as μg of quercetin equivalent·mL^−1^ and μg of quercetin equivalent·g f.w.^−1^ (for EO and plant material, respectively).

### 4.4. Cancer Cell Cultures and Experimental Setting

Three triple-negative breast cancer (TNBC) cell lines (SUM149, MDA-MB-231, MDA-MB-468) were chosen for this study. The cells belong to the basal subtype, showing ER-, PR-, HER2- immunoprofile, a high expression of proliferation marker Ki67 and epidermal growth factor receptor (EGFR+), and variable expression of basal cell marker cytokeratin 5/6 [64].

SUM149, MDA-MB-231, and MDA-MB-468 cell lines were cultured in RPMI-1640 medium (Merck KgaA) plus 10% fetal bovine serum (FBS) (Merck KgaA) at 37 °C in a humidified atmosphere containing 5% CO_2_. Before each treatment, the cells were starved for 24 h. Experimental protocols were performed on groups of cultures; samples were incubated for 48 h with dimethyl sulphoxide (DMSO) (Merck KgaA) (untreated cells, CTRL) or *L. alba* EO (treated cells) solubilized in DMSO. At the end of the treatment, the cells were washed with phosphate-buffered saline (PBS) (Merck KgaA) to remove any residues, detached with trypsin-EDTA (Merck KgaA), harvested, and then processed as reported in the following paragraphs.

### 4.5. In Vitro Tests: Proliferation and Cytotoxicity Assay

The cells were grown for 2 weeks in 25 cm^2^ flasks. After reaching 90% confluence, they were detached with trypsin-EDTA, transferred to a 96-well plate, and allowed to stand for 24 h before treatments were added. Several dilute solutions of EO were solubilized in DMSO for the tests: 0 mL·L^−1^, 0.25 mL·L^−1^, 0.5 mL·L^−1^, 1 mL·L^−1^, 2 mL·L^−1^, and undiluted EO. The solutions were prepared 24 h before the tests and stored at −20 °C until the treatments.

Cell proliferation and cytotoxicity of the treatments were analyzed using the Cell Counting Kit—8 (https://www.sigmaaldrich.com/IT/it/product/sigma/96992 (accessed on 18 December 2023)). The absorbance values were obtained, and the proliferation and toxicity percentage of the three treated cell lines were calculated and compared to the control, using a microplate reader (Spark^®^ Multimode Microplate Reader—Tecan, Männedorf, Switzerland).

### 4.6. Lactate Dehydrogenase (LDH) Activity 

Lactate dehydrogenase is an oxidoreductase enzyme that catalyzes the interconversion of pyruvate and lactate. Since LDH is a stable enzyme, it has been widely used to evaluate the presence of damage and toxicity of tissue and cells. The level of LDH in the culture medium was quantified using the Lactate Dehydrogenase Activity Assay Kit (MAK066—Sigma Aldrich (St. Louis, MO, USA)—https://www.sigmaaldrich.com/IT/it/product/sigma/mak066 (accessed on 18 December 2023) with a microplate reader (Spark^®^ Multimode Microplate Reader–Tecan, Switzerland).

### 4.7. Cells Samples Preparations 

During the preparation of samples for antioxidant and thiobarbituric acid reactive products assays, controls and treated cells with EO were detached with trypsin-EDTA, collected in 7 mL of PBS, and centrifuged for 10 min at 800× *g*. After removing the supernatant, the pellet was resuspended in 2 mL of lysis buffer (50 mM KH_2_PO_4_, 1 mM CaCl_2_, 1 mM KCl, 1 mM EDTA-Na_2_) containing a cocktail of protease inhibitors (Merck KGaA). Cells were incubated in an ice bath for 2 h and vortexed every 10 min to maximize lysis. Then, the suspensions were subjected to repeated sonication for 10 min in ice, centrifuged at 12,000× *g* for 20 min at +4° C, and the supernatants were collected and stored at −20° C for further tests.

### 4.8. 2,2-Diphenyl-1-Picryl-Hydrazyl-Hydrate (DPPH) Free Radical Assay

The antioxidant activity of EO (dissolved in DMSO), cells, and plants were tested by DPPH assay. The measurement of the DPPH radical scavenging activity was performed according to Begum et al. [65,66] with major modifications. 

A solution of 0.5 mM DPPH (Merck KGaA) in pure ethanol (Merck KGaA) was freshly prepared and kept in the dark before the test. The reaction mixture consisted of 0.5 mL of sample at different concentrations (12.5–100 mg/mL for plant samples, 12.5–100% (*v*/*v*) for tumor and essential oil samples), 3 mL of absolute ethanol and 0.3 mL of DPPH solution. Samples were incubated for 100 min in the dark at room temperature, and the absorbances were recorded at 517 nm using a spectrophotometer (VARIAN Cary 50 Bio). The mixture of 3.3 mL of ethanol and 0.5 mL of sample served as blank. The control solution was prepared by mixing 3.5 mL of ethanol and 0.3 mL of DPPH radical solution. The antioxidant activity (AA%) was determined according to the formula by Garcia et al. [67]:

(1) $$AA\%=100-\left[\frac{\left({Abs}_{sample}-{Abs}_{blank}\right)\cdot 100}{{Abs}_{control}}\right]$$

Ascorbic acid (Merck KGaA) was used as a positive control, and the results were expressed as IC_50_ value, using a linear regression method. This value is the sample concentration able to remove 50% of the DPPH free radicals. IC_50_ value of EO and cell samples was expressed as % *v*/*v* while IC_50_ value of plant samples was expressed as mg·mL^−1^.

### 4.9. Potassium Ferricyanide and Ferric Reducing Antioxidant Power (P-FRAP and FRAP) Assays 

Scavenger activity and reducing power of cells and plants were measured with FRAP and P-FRAP assay, respectively.

P-FRAP assay was based on Hue et al. [68] with modifications: a mixture consisting of 0.05 mL of sample, 0.2 mL of 1% PBS, and 0.2 mL of 1% potassium ferricyanide (*w*/*v*) (Merck KGaA) was incubated for 20 min at 50 °C. Next, 0.25 mL of 10% trichloroacetic acid (*w*/*v*) (Merck KGaA) was added. Then, 0.5 mL of supernatant was mixed with 0.5 mL of distilled water and 0.1 mL of 0.1% ferric chloride (*w*/*v*) (Merck KGaA). Samples were incubated at 37 °C for 10 min, and the absorbance was recorded at 700 nm with a spectrophotometer (VARIAN Cary 50 Bio). The scavenging activity of samples was expressed as the %, compared to the control, set at 100% activity.

FRAP assay was based on the protocols by Gohari et al. [69] and Lim and Lim [70]. FRAP reagent was prepared by adding 4.5 mL of 10 mM TPTZ (Merck KGaA) solubilized in 40 mM HCl (Merck KGaA) and 4.5 mL of 20 mM FeCl_3_ (Merck KGaA) solution to 45 mL of 0.3 M acetate buffer pH 3.6 (1:1:10 ratio). Then, 0.2 mL of sample was added to 0.4 mL of distilled water and 3.6 mL of FRAP reagent. The mixture was incubated at 37 °C for 10 min, and the absorbances were measured at 593 nm with a spectrophotometer (VARIAN Cary 50 Bio). The ferric reducing power was determined using a calibration curve, based on known concentrations of FeSO_4_-7H_2_O (y = 1.0654x − 0.0263; R^hu^ = 0.994) (Merck KGaA) and reported as mmol FeSO_4_ equivalent·L^−1^ (cell samples) and mmol FeSO_4_ equivalent·g f.w.^−1^ (plant samples).

### 4.10. Thiobarbituric Acid Reactive Products 

Lipids peroxidation of cell and plant samples were measured by thiobarbituric acid reactive products (TBARS) assay, according to Micheli et al. [71] and Kaur and Jindal [6] with modifications. Before the assay, a fresh solution of 0.5% (*w*/*v*) thiobarbituric acid (TBA) (Merck KGaA) in 20% (*w*/*v*) trichloroacetic acid (TCA) (Merck KGaA) was prepared and stored in the dark. 0.75 mL of TBA was added to 0.7 mL of sample and incubated at 50 °C for 50 min. The blank was prepared by adding the same volumes of ddH_2_O and TBA to a test tube. The samples were then placed on ice for 10 min and transferred into a glass cuvette for absorbance reading.

The sample absorbances (at 532 nm and 600 nm) were detected using the spectrophotometer (VARIAN Cary 50 Bio). TBA reactive species were expressed as malondialdehyde (MDA) equivalent, according to the following formula:

(2) $$MDAequivalent=({Abs}_{532}-{Abs}_{600})/(\varepsilon \cdot l)$$

where ε = extinction coefficient of MDA at 532 nm (155 mM^−1^ cm^−1^); l = path length of cuvette (1 cm). Data were expressed as mmol MDA equivalent·L^−1^ (cell samples) or mmol MDA equivalent·g f.w.^−1^ (plant samples).

### 4.11. Statistical Analysis

Data are expressed as mean ± standard error (SE) of two independent experiments with three replicates, unless otherwise stated. Cell data were analyzed using t-Student’s test, performed with Past 4.13. When comparing the control group to treated ones, the significance was *** *p* < 0.001; ** *p* < 0.01; * *p* < 0.05. Plant data were analyzed using one-way analysis of variance (ANOVA), performed with Past 4.13. The Tukey–Kramer method was used to assess the difference of significance among groups. All analyses were considered significant at *p* < 0.05 within each treatment group. When comparing primed groups to not primed ones, the significance was *** *p* < 0.001; ** *p* < 0.01; * *p* < 0.05.

## 5. Conclusions

The use of plant EOs is becoming even more important in many fields of biological research. However, their use is still not as common as it should be. Low concentrations of ginger grass EO have been shown to possess many beneficial properties useful both for the protection of plants against abiotic stresses, such as salinity, and for future application against human diseases like cancer. In tumor cells, *L. alba* EO showed strong cytotoxic activity, increasing ROS production and damaging the plasma membrane until cell death. Vice versa, in tomato and bean plants, the EO, used for the first time as a seed priming agent, showed an opposite effect with respect to that observed in breast cancer, protecting both crops from salt-induced damage, by enhancing antioxidant activity and inhibiting ROS production, and accordingly, improving salinity tolerance. This interesting dual performance of the EO, which depends on the treated cell type, may open a new perspective for future uses of this EO in biomedical research and plant biotechnology.

## Figures and Tables

**Table 1:** Amount of phenols and flavonoids of EO. Data are expressed as mean ± SE (*n* = 3).

	Phenols (µg Chlorogenic Acid eq.·mL^−1^)	Flavonoids (µg Quercetin eq.·mL^−1^)
*Lippia alba* EO	177.3 ± 4.2	17.2 ± 2.1

**Table 2:** Morphological parameters of plants and electrical conductivity of the soil at the end of the experiments. Each value represents mean ± SE (*n* = 6). Mean values in the column marked by different letters are significantly different within the same group (*p* < 0.05; ANOVA and Tukey–Kramer test). Significant differences to control (CTRL) are reported as * *p* < 0.05; ** *p* < 0.01; *** *p* < 0.001.

	Priming Solution	NaCl (mM)	Shoot Length (cm)	Root Length (cm)	Biomass (g)	Electrical Conductivity (dS/m)
Bean	CTRL	0	49.2 ± 1.1 ^a^	12.9 ± 2.1 ^a^	11.2 ± 3.2 ^a^	0.42 ± 0.03 ^a^
		40	33.1 ± 3.2 ^b^	11.1 ± 1.0 ^a^	11.1 ± 3.4 ^a^	0.85 ± 0.04 ^b^
		80	25.2 ± 4.4 ^b^	10.3 ± 2.1 ^a^	5.8 ± 2.4 ^a^	0.89 ± 0.03 ^b^
	*L. alba* EO	0	52.4 ± 4.3 ^a^	13.8 ± 1.2 ^a^	13.6 ± 2.7 ^a^	0.52 ± 0.08 ^a^
		40	39.1 ± 3.2 ^b^	12.7 ± 0.8 ^a^	10.7 ± 3.5 ^a^	0.55 ± 0.04 ^a^ **
		80	42.2 ± 3.2 ^ab^ **	12.4 ± 1.4 ^a^	14.0 ± 1.0 ^a *^	0.96 ± 0.05 ^b^
Tomato	CTRL	0	26.0 ± 1.3 ^a^	8.0 ± 0.5 ^a^	2.9 ± 0.1 ^a^	0.99 ± 0.02 ^a^
		160	14.1 ± 1.2 ^b^	5.0 ± 0.2 ^b^	0.7 ± 0.1 ^b^	2.37 ± 0.07 ^b^
	*L. alba* EO	0	26.0 ± 0.5 ^a^	10.0 ± 0.6 ^a^ *	3.1 ± 0.2 ^a^	1.28 ± 0.06 ^a^ *
		160	18.0 ± 0.5 ^b *^	10.0 ± 0.8 ^a^ ***	1.2 ± 0.1 ^b **^	2.91 ± 0.05 ^b^ ***

**Table 3:** Ferric reducing antioxidant power and scavenging activity of plants. Data are expressed as mean ± SE (*n* = 6). Mean values in the column marked by different letters are significantly different within the same group (*p* < 0.05; ANOVA and Tukey–Kramer test). Significant differences to CTRL are reported as * *p* < 0.05; ** *p* < 0.01; *** *p* < 0.001.

	Priming Solution	NaCl (mM)	Reducing Power (mmol FeSO_4_ eq.·g f.w.^−1^)	Scavenger Activity (%)
Bean	CTRL	0	0.0042 ± 0.0004 ^a^	100.0 ± 8.0 ^a^
		40	0.0044 ± 0.0001 ^a^	118.3 ± 7.7 ^a^
		80	0.0041 ± 0.0002 ^a^	104.4 ± 5.4 ^a^
	*L. alba* EO	0	0.0082 ± 0.0003 ^a^ ***	185.6 ± 6.1 ^a^ ***
		40	0.0063 ± 0.0004 ^b^ **	156.6 ± 4.3 ^ab^ *
		80	0.0060 ± 0.0003 ^b^ **	140.4 ± 4.8 ^b^ *
Tomato	CTRL	0	0.0041 ± 0.0003 ^a^	100.0 ± 3.4 ^a^
		160	0.0030 ± 0.0003 ^b^	16.3 ± 1.7 ^b^
	*L. alba* EO	0	0.0053 ± 0.0001 ^a^ *	158.8 ± 7.8 ^a^ ***
		160	0.0064 ± 0.0005 ^a^ ***	117.7 ± 7.3 ^b^ ***

## Data Availability

Data are contained in the present work.

## Supplementary Materials

The supporting information can be downloaded at: https://www.mdpi.com/article/10.3390/ijms25158276/s1.
