# Supplementary material for: Antioxidant Properties of Lippia alba Essential Oil: A Potential Treatment for Oxidative Stress-Related Conditions in Plants and Cancer Cells
Source: Int J Mol Sci. 2024 Jul 29;25(15):8276. doi: 10.3390/ijms25158276 (PMC11312047; doi:10.3390/ijms25158276)
Supplement: Supplementary file 1 [file ijms-25-08276-s001.zip › ijms-3095758-supplementary.pdf]

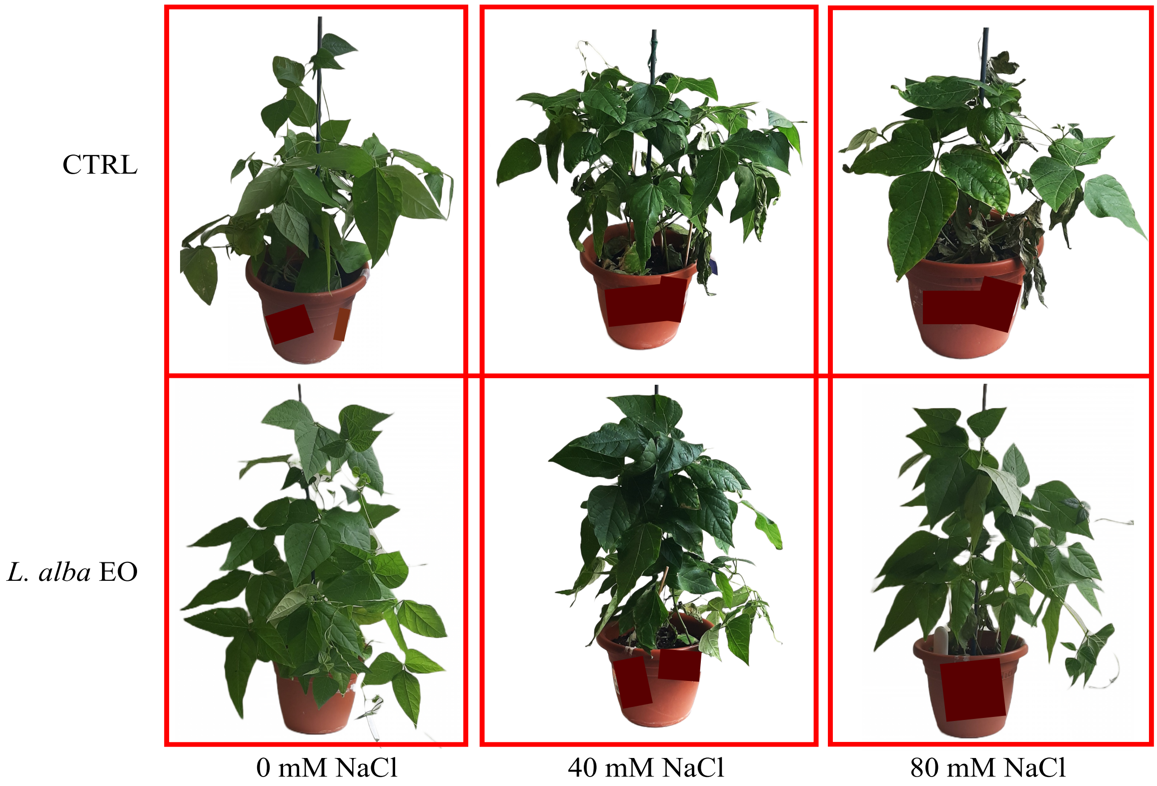

Figure S1. Not primed (CTRL) and primed (*L. alba* EO) bean plants at the end of the experiments, irrigated with 0, 40 and 80 mM NaCl.

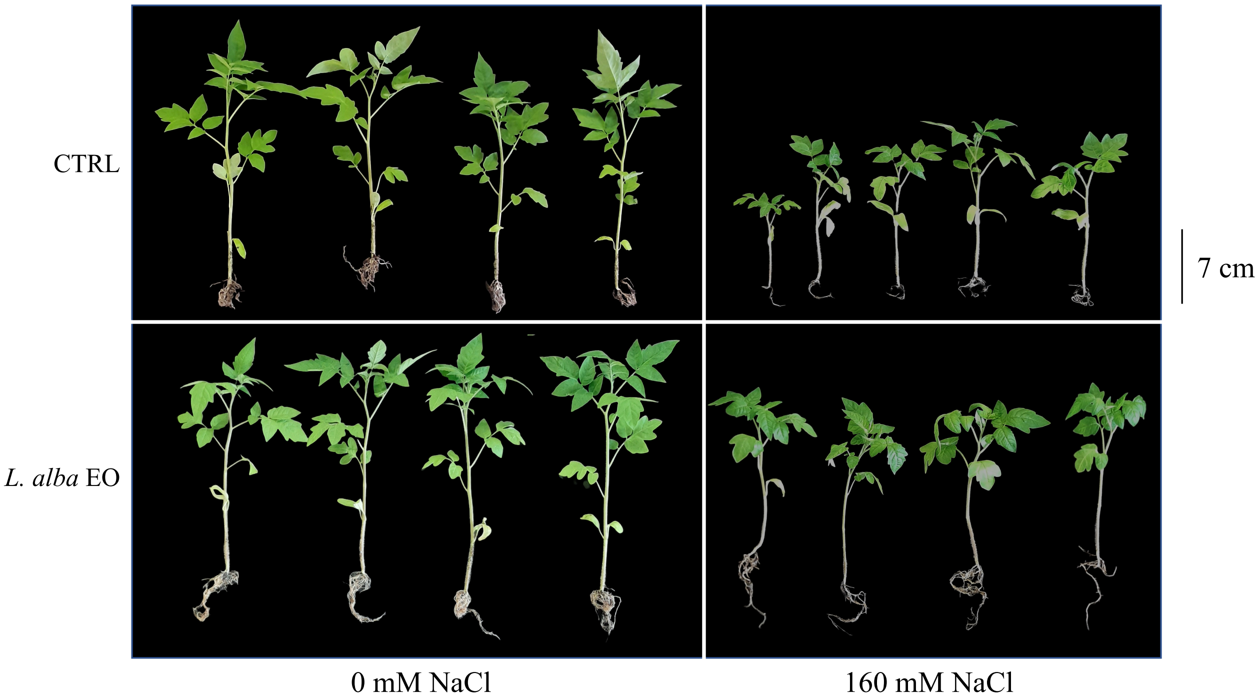

Figure S2. Not primed (CTRL) and primed (*L. alba* EO) tomato plants at the end of the experiments, irrigated with 0 and 160 mM NaCl.

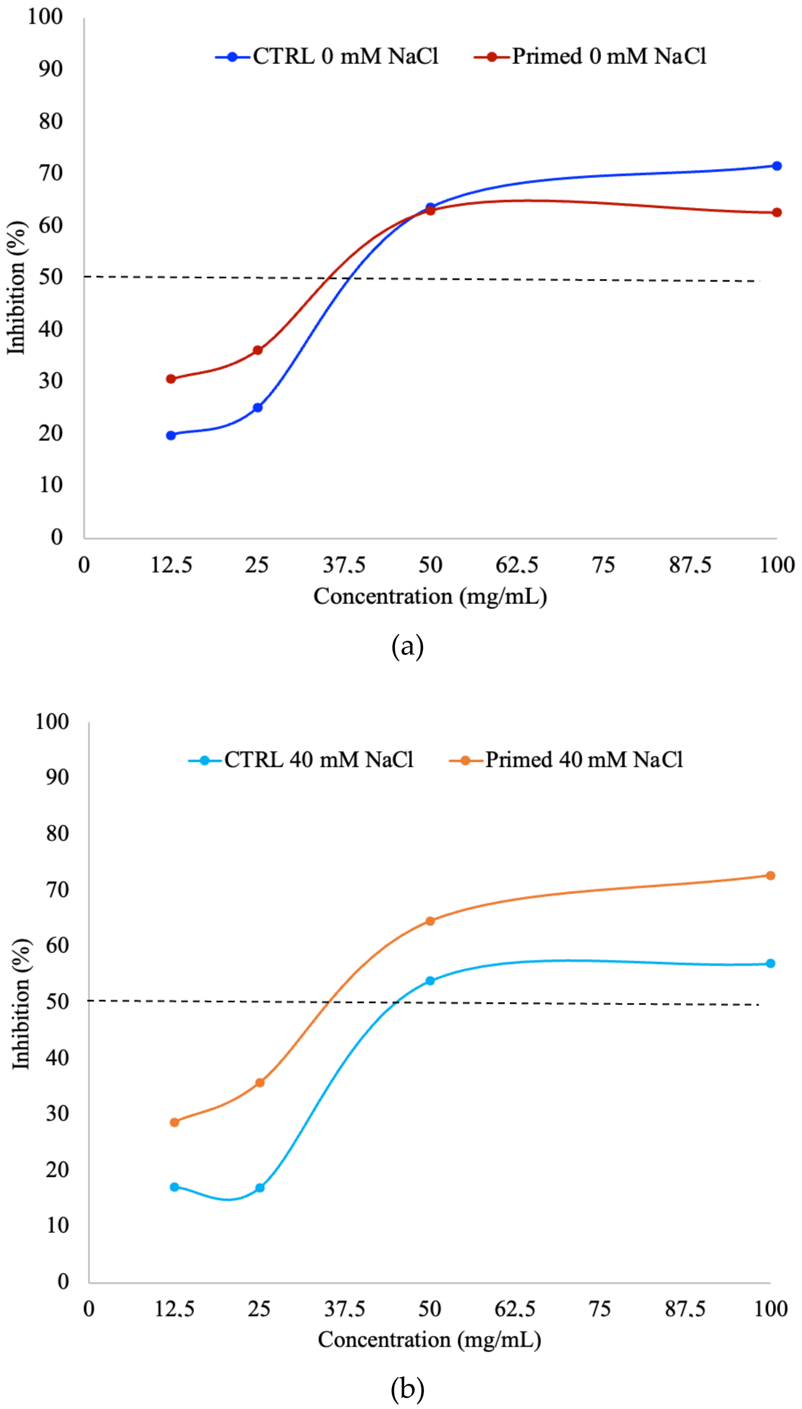

CTRL 0 mM NaCl
Primed 0 mM NaCl
Inhibition (%)
100
90
80
70
60
50
40
30
20
10
0
0
12.5
25
37.5
50
62.5
75
87.5
100
Concentration (mg/mL)
(a)
CTRL 40 mM NaCl
Primed 40 mM NaCl
Inhibition (%)
100
90
80
70
60
50
40
30
20
10
0
0
12.5
25
37.5
50
62.5
75
87.5
100
Concentration (mg/mL)
(b)

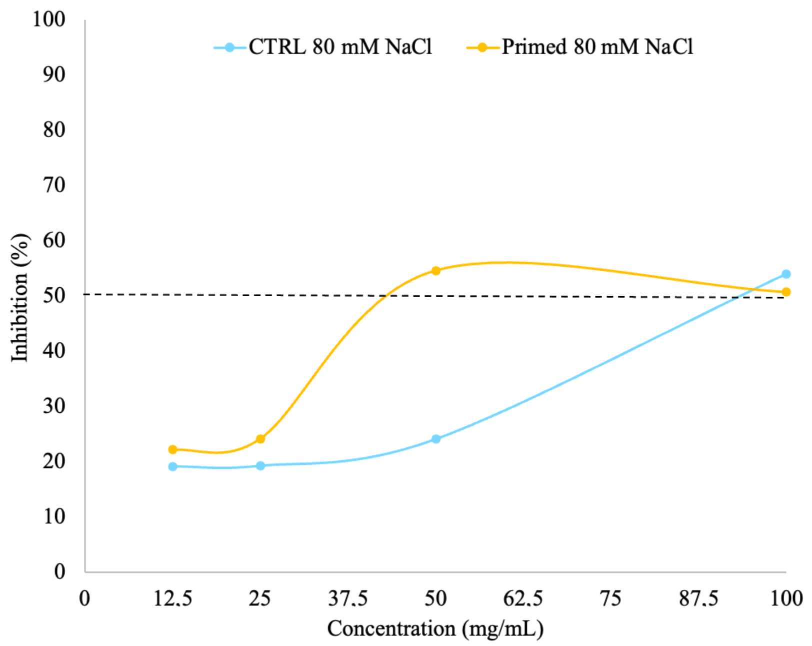

(c)

Figure S3. DPPH free radical inhibiting activity (%) at various concentration of bean leaves extract (a, b and c) expressed as mg/mL. The dotted line represents 50% of inhibition.

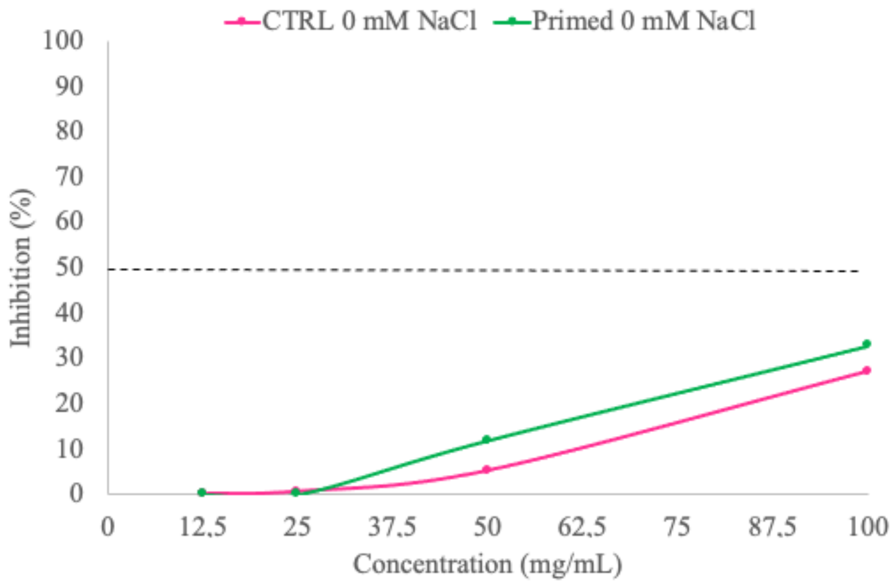

(a)

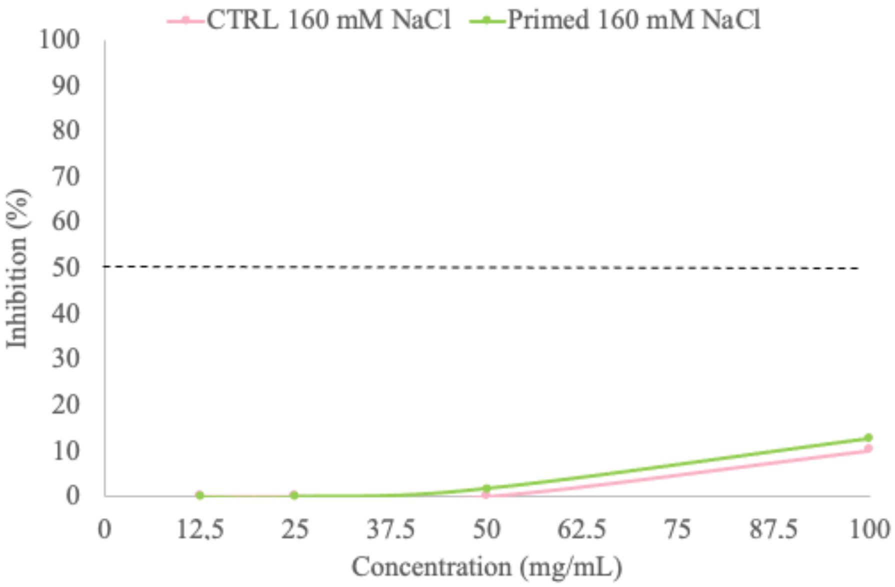

(b)

Figure S4. DPPH free radical inhibiting activity (%) at various concentration of tomato leaves extract (a and b) expressed as mg/mL. The dotted line represents 50% of inhibition.

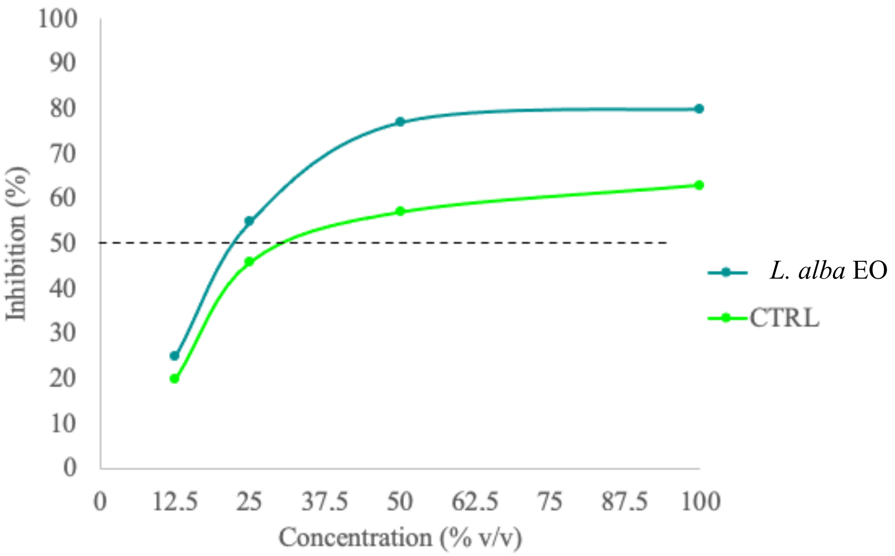

(a)

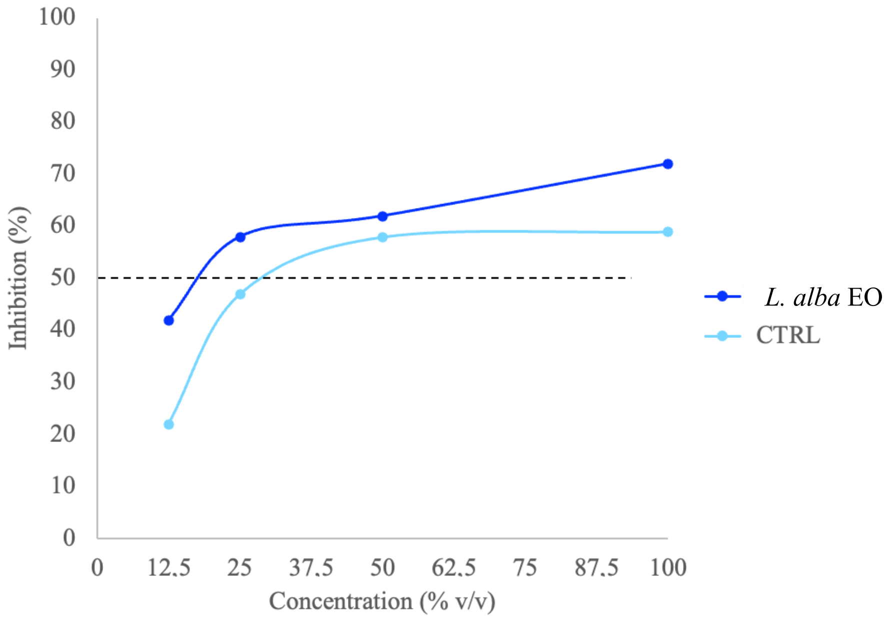

(b)

Figure S5. DPPH free radical inhibiting activity (%) at various concentration of cell extract from SUM149 (a) and MDA-MB-231 (b) expressed as % v/v. The dotted line represents 50% of inhibition.
